# An update on the use of gamma (multi)sensory stimulation for Alzheimer’s disease treatment

**DOI:** 10.3389/fnagi.2022.1095081

**Published:** 2022-12-15

**Authors:** Valerio Manippa, Annalisa Palmisano, Marco Filardi, Davide Vilella, Michael A. Nitsche, Davide Rivolta, Giancarlo Logroscino

**Affiliations:** ^1^Department of Education, Psychology and Communication, University of Bari Aldo Moro, Bari, Italy; ^2^Department of Psychology and Neurosciences, Leibniz Research Centre for Working Environment and Human Factors, Dortmund, Germany; ^3^Department of Basic Medicine, Neuroscience and Sense Organs, University of Bari Aldo Moro, Bari, Italy; ^4^Center for Neurodegenerative Diseases and the Aging Brain, University of Bari Aldo Moro at Pia Fondazione “Card. G. Panico”, Tricase, Italy; ^5^Bielefeld University, University Hospital OWL, Protestant Hospital of Bethel Foundation, University Clinic of Psychiatry and Psychotherapy and University Clinic of Child and Adolescent Psychiatry and Psychotherapy, Bielefeld, Germany

**Keywords:** 40 Hz stimulation, gamma waves, dementia, multi-sensory therapy, non-invasive brain stimulation, neuromodulation

## Abstract

Alzheimer’s disease (AD) is characterized by reduced fast brain oscillations in the gamma band (γ, > 30 Hz). Several animal studies show that inducing gamma oscillations through (multi)sensory stimulation at 40 Hz has the potential to impact AD-related cognitive decline and neuropathological processes, including amyloid plaques deposition, neurofibrillary tangles formation, and neuronal and synaptic loss. Therefore Gamma Entrainment Using Sensory stimulation (GENUS) is among the most promising approaches for AD patients’ treatment. This review summarizes the evidence on GENUS effectiveness, from animal models to AD patients. Despite the application on human is in its infancy, the available findings suggest its feasibility for the treatment of AD. We discuss such results in light of parameter improvement and possible underlying mechanisms. We finally emphasize the need for further research for its development as a disease-modifying non-pharmacological intervention.

## 1. Background

Alzheimer’s Disease (AD), the most common form of dementia, is characterized by a general cognitive decline that typically begins with episodic memory impairment and loss of functional independence (Masters et al., 2015). From a neuropathological perspective, AD is characterized by the accumulation of extracellular β-amyloid (Aβ) plaques and intracellular neurofibrillary tangles of phosphorylated tau protein (p-tau) in the brain, which initiates a neurotoxic cascade of progressive neuronal and synaptic loss in the hippocampus and surrounding medial temporal lobe (Bloom, 2014), and general brain atrophy (Pini et al., 2016). These alterations lead to imbalanced neuronal activity, reduced neuronal synchrony, and disrupted oscillatory activity at local and network levels (Mucke and Selkoe, 2012; Das et al., 2018). The available pharmacological treatments for AD show limited efficacy (Kumar and Singh, 2015); therefore, recent studies point toward alternative therapeutic avenues, such as non-invasive brain stimulation (NIBS), and multisensory stimulation targeting the restoration of abnormal brain oscillations (Menardi et al., 2021; Traikapi and Konstantinou, 2021).

A growing body of literature in the field focuses on high-frequency rhythms and their alteration (Jafari et al., 2020). Indeed, gamma oscillations (γ, > 30 Hz) exert a key role in a multitude of sensory and high-order cognitive functions (Fries, 2005; Cole and Voytek, 2017; Grent-T-Jong et al., 2018), such as episodic memory and executive functions (Carr et al., 2012; Buzsáki, 2015; Gonzalez-Perez et al., 2019), by orchestrating intra-brain communication (Ribary et al., 1991; Jefferys et al., 1996). Consistent evidence from both murine models of the disease (Yamamoto et al., 2014; Iaccarino et al., 2016) and clinical studies (Mably and Colgin, 2018) link memory and cognitive impairments to aberrant or reduced brain activity in the gamma band.

Animal models of learning paradigms provide evidence for gamma oscillations subserving the development of long-term potentiation (LTP; Bikbaev and Manahan-Vaughan, 2008), an electrophysiological correlate of learning and memory (Muller et al., 2002). LTP has been found to be affected and consistently linked to gamma rhythmicity abnormalities in AD (Li et al., 2011; Di Lorenzo et al., 2016; Koch et al., 2017). For instance, Casula et al. (2022) reported reduced gamma activity in AD patients compared with healthy controls: this decrease in frontal regions correlated with LTP–like plasticity impairment and higher cognitive decline at 24 weeks follow-up. It has been hypothesized that Aβ oligomers disrupt the excitatory/inhibitory (E/I) (i.e., glutamatergic/GABAergic) balance responsible for gamma-band oscillations, which, in turn, leads to altered hippocampal LTP activity (Lei et al., 2016). Indeed, LTP abnormalities can be prevented by modulating inhibitory GABAergic activity (Li and Selkoe, 2020).

Evidence suggests that gamma-band alterations originate from an aberrant feedback-inhibition mechanism that involves interneurons and pyramidal cells (i.e., the abovementioned glutamate-GABA dysfunction) (Rivolta et al., 2015; Palop and Mucke, 2016). Specifically, gamma-band activity is thought to arise from the activity of fast-spiking populations of GABAergic interneurons, namely parvalbumin-positive (PV^+^) cells (Rivolta et al., 2015). Restoration of PV^+^ interneurons in the hAPP mice has been found to exert a beneficial effect on gamma rhythmicity and memory (Verret et al., 2012). Noteworthy, aberrant gamma activity, particularly at 40 Hz, has been reported even before plaque formation in both rodents ad humans, suggesting that E/I imbalance and gamma alterations preceded molecular alterations in the AD neuropathological cascade (Iaccarino et al., 2016; Etter et al., 2019). This evidence has implications for the potentiality of gamma abnormality as an early biomarker of AD, as well as a therapeutic target (Goutagny et al., 2013; Mably and Colgin, 2018).

Literature suggests that 40 Hz is the most valuable target frequency (Iaccarino et al., 2016; Jones et al., 2019), with electrophysiological evidence for gamma oscillatory disruption and its modulation in AD primarily involving 40 Hz activity compared to higher frequencies (Llinas and Ribary, 1993; Jafari et al., 2020). Therefore, a promising approach receiving growing interest for the treatment of AD involves the exogenous entrainment of 40 Hz oscillations *via* transcranial alternating current stimulation (tACS; Tavakoli and Yun, 2017) or through (multi)sensory stimulation. This latter technique, usually referred to as GENUS (i.e., Gamma ENtrainment Using Sensory stimulation) relies on the optogenetic evocation of gamma oscillations (Cardin et al., 2009). This review aims to summarize the current evidence on this non-invasive technique and its effectiveness, from mice models of AD to patients.

## 2. Preclinical findings on mice models of AD

Evidence from mice models of AD points to GENUS’ potentiality in ameliorating brain pathology and cognitive symptoms. A pioneering study (Iaccarino et al., 2016) showed that both 40 Hz optogenetic and light flickering stimulation of the transgenic 5XFAD mouse model of AD, by specifically inducing fast-spiking interneuron activity in the visual cortex, can reduce Aβ levels and mitigate plaque load in the CA1 region of the hippocampus by approximately 50%. The effects reported in this study are frequency-specific, as 20 or 80 Hz stimulation did not lead to the same effects (Iaccarino et al., 2016). Similar results were reported for the TauP301S mouse model of AD, in which a reduction of p-tau accumulation in the visual cortex (in terms of number and size of tangles) was reported following daily exposure to 40 Hz flickering light stimulation for 1 h (Adaikkan et al., 2019).

Martorell et al. (2019) extended these findings to the auditory system by testing the effect of audio-visual stimulation. They showed that 1-h daily exposure to 40 Hz auditory stimulation for 1 week results in a frequency-specific effect in terms of Aβ reduction in the hippocampus and auditory cortex of the 5XFAD mice. Also, it leads to cognitive and behavioral improvement, specifically in spatial and recognition memory. The same group found that auditory stimulation reduces p-tau tangles in the P301S mice model, with effects across multiple cell types (i.e., microglia, astrocytes, and vasculature). Interestingly, providing stimulation in both sensory modalities (i.e., light and auditory), compared to the sole use of the auditory stimulation, was found to generate a microglial-clustering response (i.e., significant increases in microglia cell number and body diameter with decreased average process length), and decreased Aβ throughout the neocortex (Martorell et al., 2019).

Yao et al. (Yao et al., 2020) investigated the effect of light stimulation on the circadian clock rhythm in the APP/PS1 mice. They showed that 1-h daily 40 Hz light flickering for 30 days increased circadian locomotor activity and promoted the expression of proteins involved in the AD pathophysiological cascade (i.e., BMAL1, CLOCK, and PER2; Vanderheyden et al., 2018). They found that mice treated with 40 Hz light flicker exhibited increased gamma activity within the visual cortex. Reduced Aβ and p-tau production in the hypothalamus and restoration of electrophysiological alterations in this area were also observed (Yao et al., 2020).

Finally, Park et al. (2020) combined 40 Hz light flickering and physical exercise in the 3xTg murine model of AD to investigate their effects on learning, memory, and multiple neurobiological outcomes. They found a reduction of p-tau and Aβ in the hippocampus, together with an improvement in spatial learning, memory, mitochondrial function, and neuroplasticity in rats receiving 40 Hz light flickering. Interestingly, the combination of exercise and light flickering exposure was the most effective treatment in reducing Aβ and p-tau levels, suggesting that physical activity may represent a complementary therapy to sensory stimulation. Indeed, multiple evidence highlights physical activity efficacy in reinstating hippocampal functioning and promoting neurogenesis and plasticity (Intlekofer and Cotman, 2013).

## 3. GENUS in healthy humans

Overall, the abovementioned studies provide evidence for GENUS potential to ameliorate several components of the AD key neuropathological processes, such as amyloid plaques, neurofibrillary tangles, and neuronal and synaptic loss in mice models of AD (Homolak et al., 2018; Phan and Malkani, 2019; Pak et al., 2020). Based on these findings, the next challenge of AD research is to explore whether 40 Hz sensory stimulation can entrain gamma oscillations also in humans, specifically AD patients.

Entrainment is defined as “*synchronization to a rhythmic stream (or train) of external events*” (Thut et al., 2011). In the context of multisensory stimulation, we refer to the synchronization of endogenous brain oscillations with a sensory stimulus, particularly at a frequency of 40 Hz in the present context. Two decades ago, a study reported that auditory stimulation in healthy individuals can reset endogenous 40 Hz oscillations (Llinas and Ribary, 1993), while one decade ago, Ross et al. found that 40 Hz steady-state oscillation can be modulated or “driven” by vibrotactile stimulation (Ross et al., 2013). Despite entraining gamma oscillations were not the focus of the aforementioned studies (i.e., they did not provide gamma sensory stimulation), their findings laid the foundation for modern studies exploring 40 Hz entrainment through (multi)sensory stimulation.

Jones et al. (2019) investigated whether light stimulation, administered by an Arduino-controlled ring of ten white light LEDs with modulable frequencies and intensities, can entrain 40 Hz activity in the human brain. Specifically, electroencephalographic (EEG) brain activity was recorded in three healthy participants undergoing light stimulation at different gamma frequencies (40 Hz, 60 Hz, and 80 Hz) and intensities (high vs. low), with both eyes open and closed. Power-spectral density analyses showed that the entrainment was larger at high intensity (as compared with low intensity) 40 Hz stimulation when compared to 60 and 80 Hz, and when participants had their eyes open (as compared to closed eyes). Interestingly, the number of localizations exhibiting entrainment increased with light intensity, with the largest amplitudes in the primary visual cortex (Pz, O1, and O2 electrodes). Despite inter- and intra-individual variability, brain response to high intensity 40Hz was widespread, with 35⁓75% of the electrodes showing entrainment, and few electrodes exhibiting entrainment in temporal lobes (i.e., the most crucial area for AD pathology progression).

Recently, Lee et al. (2021) and Park et al. (2022) investigated the optimal color, intensity, and frequency of gamma light stimulus for entraining gamma activity in young and older adults, respectively. Particularly, adverse events, event-related synchronization (ERS) values (assessed through EEG), and the propagation of gamma oscillations were evaluated using different light colors, luminance intensities, and flickering frequencies from 32 to 50 Hz. As for the young adult, the authors found that red and white lights entrained gamma more effectively than green and blue lights, whereas lights of higher luminance intensities (700 and 400 cd/m^2^) entrained gamma oscillations better than lower luminance intensities (100 and 10 cd/m^2^) even though 700 cd/m^2^ resulted in more adverse effects (i.e., dazzling, asthenopia, and fatigue). In addition, entrainment following lights flickering at 34–38 Hz was stronger and more widespread (i.e., beyond the visual cortex) than that of 40–50 Hz flickering. To sum up, 400 cd/m^2^ white light flickering at 34–38 Hz was the optimal set-up to entrain gamma oscillation in young adults (Lee et al., 2021). Findings from older adults were slightly different. Specifically, 700 cd/m^2^ intensity and 32/34 Hz frequency were the optimal parameters of the flickering light stimulus to entrain gamma rhythm in parieto-occipital cortex and, by propagation, in fronto-temporal cortex with tolerable adverse effects (Park et al., 2022). These findings demonstrate that the optimal stimulation parameters to effectively induce gamma oscillations vary based on the characteristics of the entrained brain (e.g., age, neurodegeneration).

These methodological studies on gamma light flickering reported few adverse effects (e.g., asthenopia, fatigue, and dazzling) when brief stimulation was administered on healthy participants. On the other hand, long-term exposure to 40 Hz visual stimulation, and the consequent gamma entrainment within the visual cortex, poses some health-related risks for neurological patients. For instance, Hermes et al. (2017) reported that images eliciting gamma oscillations in the visual cortex are more likely to provoke seizures or pre-seizure activity in patients with photosensitive epilepsy. A possible solution could be the use of auditory stimulation. Furthermore, given the localization of auditory cortex, sound stimulation might be more effective than light stimulation in entraining gamma oscillations in the temporal lobes. Therefore, a recent study (Han et al., 2022) compared different conditions of auditory 40 Hz entrainment (i.e., sounds with sinusoidal or square waves; open-eye and closed-eye states), with EEG recorded before and after stimulation. Forty hertz sinusoidal waves with closed eyes induced the strongest gamma response in the prefrontal regions among all conditions. Interestingly, suppression of alpha rhythms followed 40 Hz square wave sounds exposure in the closed-eyes condition. Although targeting the auditory cortex, activity in the temporal lobes was not entrained by 40 Hz auditory stimulation.

Recently, Khachatryan et al. (2022) investigated if 40 Hz entrainment in healthy participants could be improved by modulating cognitive workload during stimulation sessions. The authors administered 5 min sessions of gamma light flickering to 16 participants *via* a LED matrix (FND-588XW4SM00BW35, Forge, United Kingdom). Forty hertz regular flickering light stimulation was conducted in three out of four sessions, while irregular random gamma flickering was provided in the fourth one. One out of three 40 Hz sessions was combined with a mental counting task, while another session included a visual attention task (i.e., an oddball paradigm). EEG activity was recorded before and after each GENUS session in 15 healthy participants, and *via* implanted intracranial EEG in one epilepsy patient. The authors found that 40 Hz visual flicker induced gamma entrainment in posterior brain regions (i.e., the visual cortex), as compared to the irregular condition, and regardless of the performed task. However, performing a cognitive task during the 40 Hz session improved the strength and extent of the gamma entrainment even in the temporal cortex. As compared to stimulation with no concurrent cognitive task, this condition promoted the propagation of gamma entrainment to deep areas, including the hippocampus, exerting a key role in AD neuropathology.

## 4. GENUS for AD patients’ treatment

Despite little evidence being available for sensory gamma stimulation in healthy participants, four studies explored the safety and efficacy of GENUS in clinical samples. Clements-Cortes et al. (2016) assessed the effect of 40 Hz multisensory stimulation in AD by administering Rhythmic Sensory Stimulation (RSS) (i.e., vibrotactile stimulation combined with low-frequency sound stimulation). The authors assessed the effects on gamma entrainment and cognition in AD patients at different clinical stages. Specifically, 40 Hz RSS was provided for 30 min by computer-generated low-frequency sinusoidal sound waves broadcasted through six speakers for full-body vibrotactile stimulation. Eighteen participants (6 AD patients with mild, 6 with moderate, and 6 with severe symptoms) underwent 40 Hz RSS vs. a control visual stimulation (i.e., videos of ocean waves and/or nature), each applied twice a week over 6 weeks. Outcome measures included the assessment of cognitive functioning by the St. Louis University Mental Status Test (SLUMS) (administered after each session), and the Observed Emotion Rating Scale (administered before and after the 6-weeks treatment). Behavioral observations were also conducted during each session. Forty hertz RSS led to progressive cognitive improvement compared with the control visual stimulation condition. Behavioral observations supported the quantitative findings, suggesting that 40 Hz RSS might exert a prominent clinical impact in patients with mild and moderate AD.

In Ismail et al. (2018), Ismail and co-authors administered 10 days of 40 Hz light therapy, with 2 h of daily stimulation in six Aβ-positive patients (five AD and one with Mild Cognitive Impairment—MCI). Before and after the intervention, Aβ burden was evaluated through the radiotracer C-Pittsburgh compound B (PiB) with positron emission tomography (PET; Klunk et al., 2004). Following 10 days of 40 Hz light therapy, no significant decrease of Aβ load was detected in any of the tested volumes of interest (i.e., primary visual cortex, visual association cortex, lateral parietal cortex, precuneus, and posterior cingulate), nor in the motor cortex. The authors suggested that longer treatments than those administered to transgenic AD mice may be necessary to induce amyloid reduction in humans.

Two further studies adopted a device developed by Cognito Therapeutics, Inc. (Cambridge, Massachusetts, United States) for multisensory stimulation (Cimenser et al., 2021; He et al., 2021). This device includes a handheld controller, an eye-set for visual stimulation, and headphones for auditory stimulation, to deliver 40 Hz auditory, visual, or combined audiovisual stimulation (AVS) synchronized with high temporal precision. He et al. (2021) recruited 10 patients with MCI who underwent 1-h/day of 40 Hz AVS for either 8 or 4 weeks. The primary outcomes of the study were safety, tolerability, and feasibility, which were assessed weekly throughout the trials. Levels of immune factors, Aβ and tau (assessed in cerebral spinal fluid), brain functional connectivity (based on resting-state functional magnetic resonance imaging—fMRI), and EEG data were also collected before, during and after the treatment. No cognitive and behavioral outcomes were assessed. Participants reported the stimulation to be tolerable, with only one participant not tolerating flicker light stimulation, and showed a high rate of adherence (95.5% on average), and compliance (i.e., 90% of the patients decided to participate in an open-label extension of the trial). Few participants experienced minor adverse events (e.g., dizziness, tinnitus, headache, and hearing loss). EEG recordings indicated that neural activity was effectively entrained at 40 Hz during each session. However, no significant increase of gamma oscillations emerged following treatment compared with baseline. On the contrary, a decrease of gamma power oscillations in the left occipital region was found after 8 weeks of 40 Hz AVS. Functional connectivity between the posterior cingulate cortex (PCC) and precuneus, and between the PCC and the medial prefrontal cortex nodes of the default mode networ, which is weakened in AD (Yi et al., 2015; Badhwar et al., 2017), increased after 8 weeks of 40 Hz stimulation. No significant changes emerged in Aβ and p-tau levels, despite analyses of immune profiles showing a trend toward downregulation of immune factors. This suggests that long-term flicker therapy may attenuate potentially harmful cytokines involved in the activation of microglia and astrocytes.

Preliminary data are available from a clinical trial investigating AVS vs. sham (i.e., placebo) induction of cortical gamma oscillations in mild to moderate AD patients *via* the Cognito Device (Overture, NCT03556280; Cimenser et al., 2021). Participants in the active treatment group received 1-h daily sessions of 40 Hz AVS with eyes closed over 6 months, while those in the sham group were exposed to AVS randomly ranging from 1 to 100 Hz, designed to not evoke cortical 40 Hz steady-state oscillations (Herrmann, 2001). Patients’ nighttime activities were monitored with continuous actigraphy recordings, while their functional abilities were measured *via* the Alzheimer’s Disease Cooperative Study–Activities of Daily Living (ADCS-ADL) scale. Results demonstrated that all participants well tolerated the treatment. Patients receiving 40 Hz stimulation showed a reduction in nighttime “active” periods and maintained their functional abilities over the treatment duration. Conversely, participants in the sham group exhibited sleep quality deterioration and a decline in ADCS-ADL scores. These findings confirm the feasibility, safety, and therapeutic potential of 40 Hz AVS for AD treatment.

Another study, available as a preprint, investigated the safety, compliance, and entrainment capacity of the same AVS device (Clinical trial: NCT 04042922; Chan et al., 2021c). Specifically, AVS effects on brain structure, sleep activity, and cognitive functions were evaluated in cognitively unimpaired participants (13 young and 12 older) and 16 patients with AD. Authors showed that 40 Hz AVS for 3 months effectively induced gamma entrainment across multiple brain regions in all participant groups, as compared to placebo treatment (e.g., constant light and white noise) which was ineffective. Treatment entrained gamma activity in cortical and subcortical regions, including those that visual or auditory stimulation alone did not entrain in previous studies. Moreover, 40 Hz AVS proved to be a safe technique (i.e., did not trigger epileptiform activity even in a sample of patients with epilepsy, and did not cause any severe adverse effects in AD and controls). Interestingly, patients receiving 40 Hz AVS showed less ventricular enlargement, together with greater stability of the hippocampal size compared to the placebo group. Functional connectivity increased in both the default mode network and medial visual network after 3 months of 40 Hz AVS stimulation. Also, circadian rhythmicity (i.e., intradaily variability and interdaily stability), which is known to be disrupted in AD patients (van Someren et al., 1996), improved following the treatment. As for the cognitive outcomes, performance at the face-name association delayed recall test improved after 40 Hz AVS, but not following placebo interventions.

## 5. Conclusions and future perspectives

Targeting gamma rhythms in AD is receiving growing interest as a therapeutic option due to available evidence for an involvement of high-frequency oscillations in AD pathology, and the promising entrainment effect emerged in both mouse models and patients (Traikapi and Konstantinou, 2021; Chan et al., 2021b). Sophisticated and multimodal tools for brain rhythm entrainment are now being developed (Menardi et al., 2021). Despite research on the effects of GENUS is in its infancy, the available studies suggest that 40 Hz multisensory stimulation could be a safe, feasible, and tolerable approach able to entrain gamma oscillations. These features point to its potential future application in both clinical and home-based settings, although more and larger studies for routine application implementation still needed.

Although some evidence shows that GENUS can improve cognitive functioning and circadian patterns, further investigations should be pursued to ascertain the potential and effectiveness of 40 Hz (multi)sensory stimulation for AD treatment. The wide variability of sensory stimulation protocols, including light or auditory stimulation, RSS, and AVS, the number of sessions, and assessed outcomes in the literature make it not possible to draw firm conclusions at present. Multiple preliminary data, published as conference papers, suggest positive effects of 40 Hz sensory stimulation in healthy older adults (Davis et al., 2022), MCI (Hempel et al., 2021), and AD patients (Suk et al., 2020; Boasso et al., 2021; Williams et al., 2021; Chan et al., 2021a). These preliminary data consistently show that daily long-term (up to 6 months) 40 Hz light and/or sound stimulation treatment is well-tolerated and can be safely administered also at home. Compared to placebo interventions, (multi)stimulation seems to prevent cognitive decline and improve circadian patterns in both healthy and clinical populations. Nevertheless, as compared to mice models, to date no evidence is available about 40 Hz (multi)sensory stimulation effects on neuropathological biomarkers (Aβ and p-tau) in patients.

**Figure 1:**
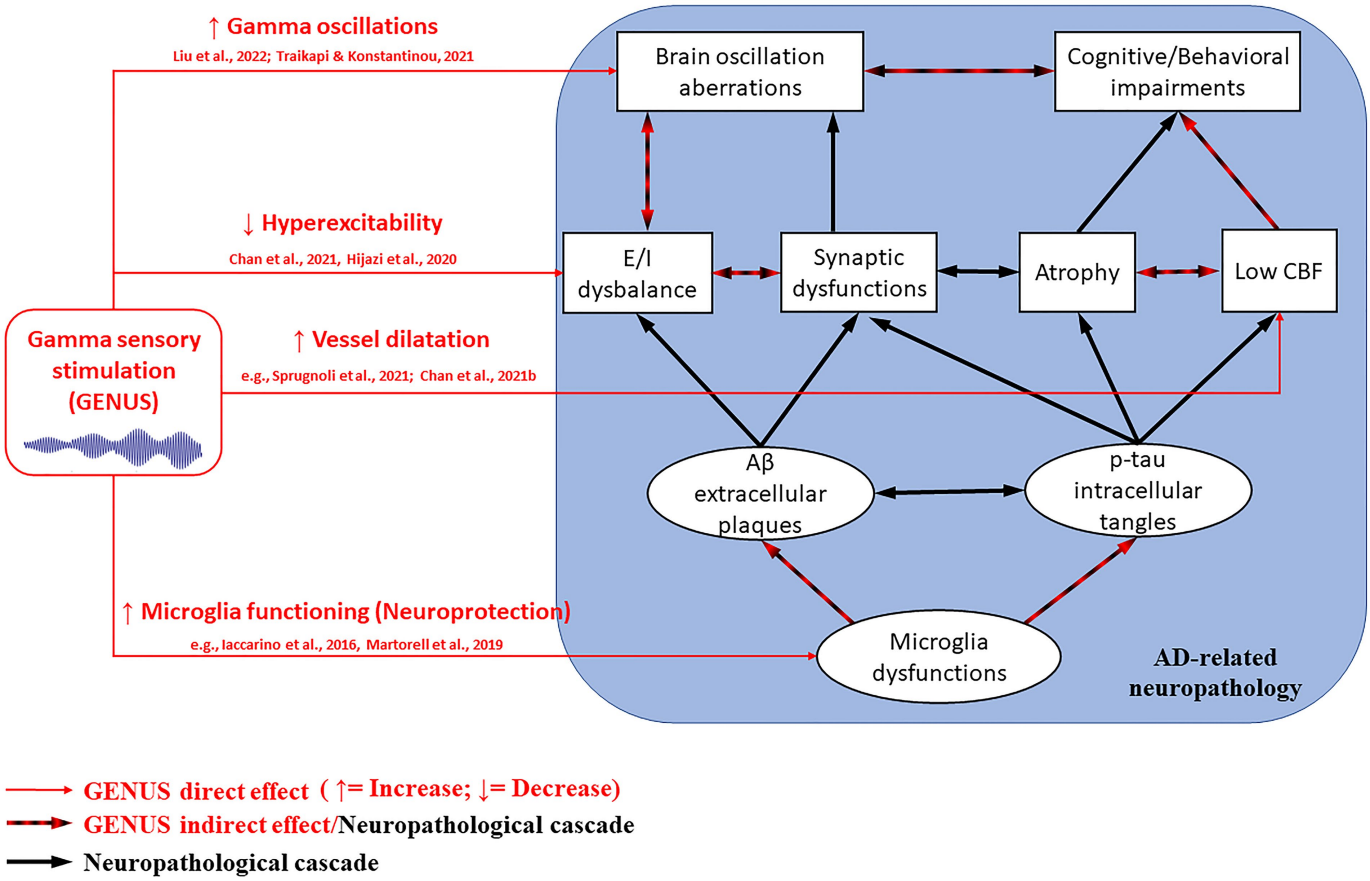
GENUS mechanisms on the AD-related neuropathological cascade (within the blue square). GENUS can directly affect (red arrows) gamma oscillations, excitatory/inhibitory (E/I) balance, cerebral blood flow (CBF), and morpho-functional properties of microglia. In turn, such improvements can indirectly impact (black and red arrows) brain atrophy process, amyloid-beta (Aβ) plaques, and neurofibrillary phosphorylated-tau (p-tau) tangles formation, thus cognitive and behavioral manifestations. The strongest effects derive from combining visual and auditory stimulation, rather than using a single modality.

GENUS’s mechanisms of action (see Figure 1) have not been well understood yet, and its impact on AD-related neuropathology needs to be further clarified. It was suggested that 40 Hz gamma stimulation in mice models attenuate AD-related pathology (i.e., Aβ, p-tau, synapses, connectivity, and cognitive function loss) by inducing glial responses promoting neuroprotection. Gamma stimulation in humans seems to improve brain vessel dilatation, and thus, cerebral blood perfusion in AD patients (Sprugnoli et al., 2021; Chan et al., 2021b). On the other hand, the observed improvements in patients’ cognitive functions obtained by neuromodulation have been attributed to the enhancement and integration of information flow. Indeed, the entrainment of gamma activity has the potential of reversing the progressive decrease of gamma over theta oscillations reported in MCI and dementia patients (Moretti et al., 2011; Musaeus et al., 2020). At the cellular level, gamma entrainment can potentially impact large-scale activity of fast-spiking PV^+^ interneurons (i.e., from visual/auditory areas to deeper brain regions, such as the hippocampus; Catani et al., 2003; Kanwisher and Dilks, 2013). Indeed, it has been hypothesized that the beneficial effects of GENUS originate from the positive effect of gamma entrainment on excitation/inhibition imbalance, as it could prevent neurochemical alterations associated with Aβ (i.e., hyperexcitability) and induce neuroprotective mechanisms (e.g., microglia activation; Hijazi et al., 2020; Chan et al., 2021a, b, c).

Longitudinal studies with larger sample sizes would shed light on methodological uncertainties and the actual therapeutic perspectives. What is the best 40 Hz sensory stimulation technique to improve gamma oscillation in AD patients? To what extent can GENUS improve cognitive functioning and neuropathology markers of AD? Does GENUS represent a valuable alternative to pharmacological interventions for AD? These are points that future studies should address. At present, two randomized controlled trials evaluating the long-term effects of AVS on AD symptoms and neuropathology are ongoing (NCT04042922, NCT03556280). Future research will also benefit from the remarkable technological progress in the development of devices for multisensory stimulation.

## Author contributions

VM: conceptualization. VM and AP: writing—original manuscript. MF, DV, MAN, DR, and GL: writing—review and editing for important intellectual content. DR and GL: supervision. All authors contributed to the article and approved the submitted version.

## Funding

This work has been supported by the founding of Regione Puglia and CNR for Tecnopolo per la Medicina di Precisione. DR no. 2117 of 21.11.2018 (CUPB84I18000540002)–C.I.R.E.M.I.C. (Research Center of Excellence for Neurodegenerative Diseases and Brain Aging)–University of Bari “Aldo Moro.” VM was supported by the project “Neuromodulatory interventions in the early stages of Alzheimer’s disease: Neurophysiological, cognitive, and computational aspects” co-funded by the European Union - FSE-REACT-EU, PON Research and Innovation 2014–2020, DM1062/2021 (CUP - H95F21001430006). MN was supported by the project “Neurotwin” founded by the Europe Union’s Horizon 2020 research and innovation program under grant agreement No. 1010177116.

## Conflict of interest

The authors declare that the research was conducted in the absence of any commercial or financial relationships that could be construed as a potential conflict of interest.

## Publisher’s note

All claims expressed in this article are solely those of the authors and do not necessarily represent those of their affiliated organizations, or those of the publisher, the editors and the reviewers. Any product that may be evaluated in this article, or claim that may be made by its manufacturer, is not guaranteed or endorsed by the publisher.
